# Robot-Assisted Laparoscopic Hiatal Hernia Repair in a Patient With Right Aortic Arch: Implications for Surgical Planning and Symptom Resolution

**DOI:** 10.7759/cureus.81966

**Published:** 2025-04-09

**Authors:** Alexandria K Howard, Paul Nordness

**Affiliations:** 1 General Surgery, Edward Via College of Osteopathic Medicine, Auburn, USA; 2 General Surgery, Gadsden Regional Medical Center, Gadsden, USA

**Keywords:** congenital vascular anomaly, paraesophageal hiatal hernia, right aortic arch (raa), right-sided aortic arch, robotic hiatal hernia

## Abstract

A right aortic arch (RAA) is a rare vascular anomaly that is often asymptomatic but may present challenges during foregut surgery depending on its branching pattern and course. We present the case of a 54-year-old female with a known RAA and gastroesophageal reflux disease (GERD) refractory to maximal medical therapy. She underwent robot-assisted laparoscopic paraesophageal hiatal hernia repair. Preoperative CT imaging confirmed a right-sided descending aorta crossing midline at T11 without evidence of extrinsic esophageal compression. Surgical precautions included careful preoperative planning and intraoperative dissection to avoid vascular injury due to the aberrant anatomy. The patient recovered uneventfully and reported resolution of reflux symptoms at follow-up, confirmed by symptom assessment and improvement in diet tolerance. This case underscores the need to consider vascular anomalies when planning surgical intervention for GERD, especially in refractory cases. It highlights the role of robotic-assisted surgery in safely managing complex anatomy.

## Introduction

A right aortic arch (RAA) is a rare anatomical variant in which the aortic arch traverses the right main bronchus and descends to the right of the thoracic vertebrae. The estimated prevalence is between 0.01% and 0.1% in the general population [1]. This anomaly results from the persistence of the right dorsal aorta and regression of the left dorsal aorta during embryologic development [2]. Normally, the right dorsal aorta regresses, allowing the left dorsal aorta to form a left-sided aortic arch and descending aorta. Recognition of RAA variants is critical in foregut surgery due to potential implications for esophageal symptoms, vascular compression, and intraoperative planning.

Most RAAs are asymptomatic and discovered incidentally. However, depending on the branching pattern and position of the descending aorta, RAA may contribute to esophageal compression or complicate hiatal dissection. Edward’s classification defines three primary subtypes. The most common is RAA with an aberrant left subclavian artery (60% of cases), which may course posterior to the esophagus and, in rare cases, arise from a Kommerell diverticulum. This aneurysmal dilation may compress mediastinal structures or rupture [3,4]. While relevant in symptomatic cases, our patient did not exhibit an aberrant subclavian artery or Kommerell diverticulum.

Another common variant is an RAA with mirror-image branching of the arch vessels [3]. This pattern is strongly associated with cyanotic congenital heart disease, particularly tetralogy of Fallot and truncus arteriosus [5]. In this configuration, the left innominate artery, right common carotid artery, and right subclavian artery arise from the right-sided arch [3]. Unlike other variants, this pattern rarely causes compression of the trachea or esophagus.

A less common subtype is an RAA with isolation of the left subclavian artery [3]. In this anomaly, the left subclavian artery is not a direct branch of the aortic arch but instead connects to the left pulmonary artery via a left ductus arteriosus [6]. This abnormal vascular connection can lead to congenital subclavian steal syndrome or vertebrobasilar insufficiency [6] and is often associated with complex congenital heart defects, including tetralogy of Fallot [3].

This report presents a case of RAA in a patient with gastroesophageal reflux disease (GERD) refractory to maximal medical therapy who underwent robot-assisted laparoscopic hiatal hernia repair. We discuss the relevance of her vascular anatomy in the context of her symptoms and describe how it influenced surgical decision-making.

## Case presentation

A 54-year-old Caucasian female with a history of GERD with esophagitis, paraesophageal hiatal hernia (PEH), RAA, peptic ulcer disease, type 2 diabetes with neuropathy, and morbid obesity (BMI 40.1) presented for surgical management of refractory acid reflux. Despite maximal medical therapy, she reported persistent heartburn, regurgitation, postprandial dysphagia, and bloating. She denied vomiting, diarrhea, melena, hematochezia, chest pain, abdominal pain, or dysphagia to liquids. Her RAA had been previously identified incidentally on imaging, and she had no history of congenital heart disease.

A prior esophagogastroduodenoscopy demonstrated a large PEH, likely the primary cause of her symptoms. Although no formal pH monitoring or esophageal manometry had been performed, the combination of persistent symptoms and endoscopic findings supported the diagnosis of medically refractory GERD. On exam, she was morbidly obese with a soft, non-tender abdomen and no guarding. There were no signs of cyanosis or edema, and peripheral pulses were equal bilaterally.

**Figure 1:**
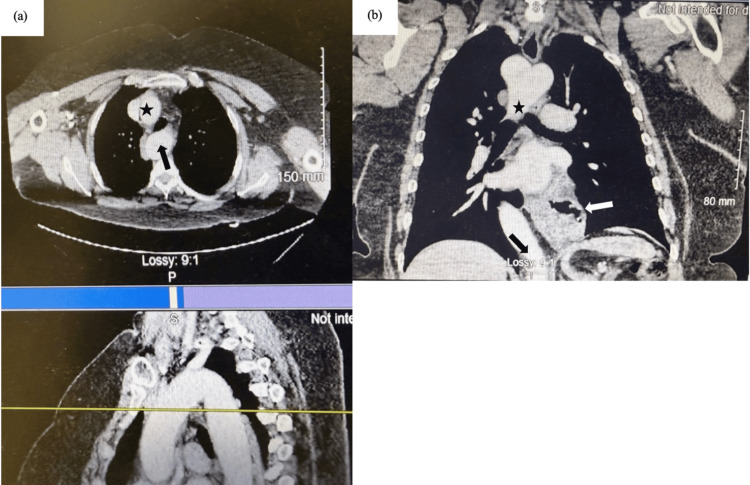
CT chest of patient with RAA and PEH (a) Ascending aorta (star) and RAA (arrow) traversing the right main bronchus. (b) Right-sided descending aorta (star) before crossing midline at the level of the diaphragm (black arrow). PEH extending into the thoracic cavity (white arrow). CT: computed tomography, RAA: right aortic arch, PEH: paraesophageal hiatal hernia

Given the presence of an RAA, a thorough preoperative imaging review was undertaken to assess for vascular compression or anatomical distortion. CT imaging confirmed a right-sided descending thoracic aorta crossing midline at T11, without evidence of a Kommerell diverticulum, aberrant subclavian artery, or extrinsic esophageal compression (Figure 1A). A PEH was seen extending above the diaphragm (Figure 1B). Her aortic anomaly was therefore deemed incidental to her reflux symptoms but posed unique surgical considerations.

The presence of the RAA altered expected retroesophageal anatomy, increasing the risk of vascular injury during hiatal dissection. No changes were required in standard trocar placement; however, the intraoperative strategy was adjusted to include cautious medial-to-lateral dissection at the hiatus and close attention to the posterior crural area, where the aorta’s proximity required meticulous technique. Robotic assistance enhanced visualization and precision in these areas. Her obesity further increased technical difficulty, limiting exposure and requiring low-pressure pneumoperitoneum with careful retraction to maintain safe working planes.

The patient underwent robot-assisted laparoscopic hiatal hernia repair without complication. Postoperatively, she recovered uneventfully and reported resolution of reflux symptoms at her follow-up visit. Her case highlights the importance of preoperative identification of vascular anomalies that, while not directly symptomatic, can significantly influence operative planning and intraoperative risk mitigation in foregut surgery.

## Discussion

A hiatal hernia occurs when a portion of the stomach prolapses through the esophageal hiatus into the thoracic cavity. As seen in our patient, a PEH results from continuous stretching of the phrenoesophageal ligament [7]. This ligament normally anchors the lower esophagus and maintains gastroesophageal competence [8]. Excess intra-abdominal pressure from obesity, aging, prior abdominal surgery, or straining can lead to lateral traction on the phrenoesophageal membrane and disruption of the lower esophageal sphincter (LES) [7]. As the LES migrates proximally into the negative pressure environment of the thoracic cavity, the pressure gradient at the gastroesophageal junction (GEJ) diminishes, increasing the risk of gastroesophageal reflux [7]. In this patient, the combination of morbid obesity and a large PEH likely contributed to persistent reflux symptoms despite medical therapy, supporting the decision for surgical repair to restore anatomic positioning of the GEJ and improve symptom control.

Most symptomatic PEHs require surgical repair to prevent life-threatening complications such as volvulus, strangulation, incarceration, or perforation [9]. Minimally invasive approaches, particularly laparoscopy, are considered the gold standard for PEH repair [9]. With or without robotic assistance, laparoscopy facilitates precise dissection of the esophageal hiatus and crura. However, controversy exists regarding procedural details, including Nissen versus partial fundoplication, reinforcement mesh use, and the role of anterior gastropexy [9]. The optimal surgical approach depends on patient anatomy and surgeon preference. In this case, the minimally invasive approach was chosen due to the patient’s obesity and to minimize recovery time, while allowing for better visualization and precision given her complex anatomical variation.

Our patient underwent a robot-assisted laparoscopic PEH repair with fundoplasty and biologic mesh reinforcement. Meticulous dissection allowed for safe reduction of the herniated contents while preserving the esophagus, vagal nerves, and displaced aorta. Posteriorly, the esophagus was mobilized from the aorta without injury, as the aorta remained visible and in its normal anatomical position. Phasix mesh (Becton, Dickinson and Company, Franklin Lakes, NJ, USA) was placed on the anterior esophageal hiatus and secured to the diaphragm with mattress sutures, with additional reinforcement of the crura bilaterally. The angle of His was reconstructed, and an anterior fundoplication was performed. Upper endoscopy confirmed a widely patent esophagus and GEJ at 40 cm, with intact fundoplication and no evidence of leak or bleeding.

The patient tolerated the procedure well, with no immediate postoperative complications. Postoperative management includes aggressive antiemetic therapy to prevent nausea and vomiting, as increased intra-abdominal pressure from retching can disrupt the repair [10]. Once nausea resolves, patients advance to a clear liquid diet, typically within the first 24-48 hours. If tolerated, they transition to a “post-esophageal surgery” diet of soft or pureed foods for approximately two weeks [10]. In the absence of nausea, vomiting, or dysphagia, they gradually resume a regular diet. At the two-week follow-up, persistent dysphagia may necessitate extending the pureed diet for up to six weeks, with further evaluation if symptoms persist [10]. Patients are counseled on strict dietary adherence, including eating small meals, chewing thoroughly, eating slowly, and maintaining an upright position after meals to optimize surgical outcomes.

## Conclusions

This case highlights the importance of identifying vascular anomalies such as an RAA in patients undergoing foregut surgery. While the RAA did not directly contribute to the patient's symptoms, it significantly influenced preoperative planning. Additional imaging was obtained to characterize the aortic course and exclude esophageal compression or associated anomalies. Although trocar placement did not require alteration, intraoperative dissection near the hiatus was modified to account for the aorta’s atypical position. Particular caution was taken during posterior crural dissection to avoid vascular injury. Robot-assisted laparoscopic repair was especially beneficial due to enhanced three-dimensional visualization and instrument precision, which facilitated safe dissection near the aberrant vasculature. A multidisciplinary team contributed to comprehensive care, including radiology for detailed imaging interpretation and gastroenterology for preoperative endoscopic evaluation. This case reinforces the value of collaborative planning and tailored surgical strategies for patients with complex anatomy.
